# Proportional Recovery After Stroke: Addressing Concerns Regarding Mathematical Coupling and Ceiling Effects

**DOI:** 10.1177/15459683231177598

**Published:** 2023-06-02

**Authors:** Benjamin Chong, Alan Wang, Cathy M. Stinear

**Affiliations:** 1 Department of Medicine, The University of Auckland, Auckland, New Zealand; 2 Centre for Brain Research, The University of Auckland, Auckland, New Zealand; 3 Auckland Bioengineering Institute, The University of Auckland, Auckland, New Zealand; 4 Centre for Medical Imaging, The University of Auckland, Auckland, New Zealand

**Keywords:** stroke, proportional recovery, statistics, recovery, modeling, impairment

## Abstract

Baseline scores after stroke have long been known as a good predictor of post-stroke outcomes. Similarly, the extent of baseline impairment has been shown to strongly correlate with spontaneous recovery in the first 3 to 6 months after stroke, a principle known as proportional recovery. However, recent critiques have proposed that proportional recovery is confounded, most notably by mathematical coupling and ceiling effects, and that it may not be a valid model for post-stroke recovery. This article reviews the current understanding of proportional recovery after stroke, discusses its supposed confounds of mathematical coupling and ceiling effects, and comments on the validity and usefulness of proportional recovery as a model for post-stroke recovery. We demonstrate that mathematical coupling of the true measurement value is not a real statistical confound, but rather a notational construct that has no effect on the correlation itself. On the other hand, mathematical coupling does apply to the measurement error and can spuriously amplify correlation effect sizes, but should be negligible in most cases. We also explain that compression toward ceiling and the corresponding proportional recovery relationship are consistent with our understanding of post-stroke recovery dynamics, rather than being unwanted confounds. However, while proportional recovery is valid, it is not particularly groundbreaking or meaningful as previously thought, just like how correlations between baseline scores and outcomes are relatively common in stroke research. Whether through proportional recovery or baseline-outcome regression, baseline scores are a starting point for investigating factors that determine recovery and outcomes after stroke.

## Introduction

Recovery and outcomes after stroke exhibit considerable inter-individual variability, often analyzed using linear regression modeling. Many studies to date have found correlations between baseline and outcome scores. Similarly, some studies have found correlations between baseline severity and recovery, where greater baseline severity corresponds to greater recovery, though not enough to result in better outcomes. This concept, known as proportional recovery, is commonly demonstrated for recovery from upper limb motor impairment, but has also been observed in other post-stroke impairments. Thus, several authors have suggested that proportional recovery represents spontaneous biological recovery after stroke.^1-3^ However, various concerns have been raised regarding proportional recovery’s potential confounds and its validity as a model for post-stroke recovery. This article gives an overview of proportional recovery, discusses its proposed confounds, and makes some recommendations for the field.

## Proportional Recovery

Outcome is the absolute performance measured at some endpoint, while recovery refers to change in performance over time, calculated as the difference in score between 2 timepoints. Proportional recovery refers to the apparent group-level linear relationship between baseline impairment and spontaneous recovery from impairment after stroke, usually measured at 3 and 6 months post-stroke. The standard formula for proportional recovery is given by equation (1), where *X* represents baseline scores and *Y* represents outcome scores.

The proportionality between recovery (*Y* – *X*) and baseline impairment (max score – *X*) is represented by the slope *β*_1_, with *c*_1_ as the intercept. Many studies define proportional recovery as 70% recovery proportionality, particularly for the Fugl-Meyer Upper Extremity (FM-UE), however, this article shall discuss proportional recovery more generally as any correlation between baseline impairment and recovery, regardless of the value of *β*_1_. This is because *β*_1_ is also dependent on the scale used, so a slope of 70% should not be considered generalizable to all measurement scales. Proportional recovery can also be expressed as the correlation between baseline scores (*X*) and recovery (*Y* – *X*), where the slope is equal to –*β*_1_.^4,5^

Proportional recovery after stroke was first described in 2008 by Prabhakaran et al,^6^ who found that the strongest correlate of recovery in the FM-UE at 3 and 6 months post-stroke was baseline FM-UE impairment. Most patients recovered approximately 70% of their baseline impairment, while some outlier patients had severe baseline impairments and poor recoveries. Proportional recovery in the FM-UE has since been reproduced using various methods. Some studies reevaluate the recovery proportion for their study sample,^1,7,8^ while other studies predefine a recovery proportion of 70%,^9-12^ or both.^13^ Overall, most patients recover about 60% to 80% of their baseline FM-UE impairment within 3 to 6 months post-stroke. People who fit this group-wise relationship are known as fitters, while those who do not fit the relationship are known as non-fitters, generally have severe baseline impairments, and experience poor recoveries well below 70%.^9,11,13^ However, fitters can also have severe baseline impairments, indicating that this alone does not preclude proportional recovery.

### Distinguishing Fitters and Non-Fitters

As baseline impairment alone is insufficient to distinguish fitters from non-fitters, several biomarker-based approaches have been investigated for this purpose. Byblow et al^1^ and Stinear et al^8^ found that proportional recovery in the FM-UE at 3 and 6 months post-stroke applied to the group of patients with upper limb motor evoked potentials, a transcranial magnetic stimulation indicator of preserved corticospinal tract function. This was true even for patients with severe baseline impairments. Patients with no upper limb motor evoked potentials had poor recoveries that did not correlate with baseline impairment. The presence or absence of a motor evoked potential predicted fitters and non-fitters with 85% and 91% accuracy, respectively.^1^

Buch et al^10^ and Guggisberg et al^13^ found that early after stroke, non-fitters overall had more asymmetric fractional anisotropy in the corticospinal tract than fitters, indicative of disrupted white matter structural integrity. Fractional anisotropy asymmetry in the corticospinal tract at 2 weeks post-stroke classified fitters and non-fitters with 80% accuracy.^10^ Liu et al^11^ found no differences in fractional anisotropy between fitters and non-fitters, but observed that non-fitters generally had lower mean diffusivity and local diffusion homogeneity than fitters in various subcortical regions early after stroke, also indicative of disrupted white matter structural integrity. Recently, Liu et al^12^ found that compared to fitters, non-fitters had reduced structural volume of various regions, such as the corticospinal tract and cerebellum, and that a combination of FM-UE scores and whole brain volumes at baseline could classify fitters and non-fitters with 88% accuracy. Using electroencephalography, Guggisberg et al^13^ found that compared to fitters, non-fitters had lower overall beta-band coherence, a marker of functional connectivity, between ipsilesional ventral premotor cortex and primary motor cortex at 2 to 4 weeks after stroke.

### Proportional Recovery From Other Impairments

While most proportional recovery research has focused on the FM-UE, studies have also demonstrated proportional recovery relationships for lower limb motor impairment,^2,14^ sensation,^7^ aphasia,^5,15^ visuospatial neglect,^3,15^ memory,^16^ attention,^16^ and resting motor threshold.^1^ Fitters and non-fitters have been identified for visuospatial neglect,^3,15^ and inconsistently for recovery from aphasia^5,15^ and lower limb impairment.^2,14^ Similar to proportional recovery in the FM-UE, non-fitters in other neurological domains generally have severe baseline impairments in that domain, however, not all patients with severe baseline impairments are non-fitters.^2,3,15^ Marchi et al^15^ reported no differences in age, sex, lesion volume, or therapy dose between fitters and non-fitters for proportional recovery from visuospatial neglect and aphasia. Winters et al found that non-fitters for neglect were also non-fitters for FM-UE, and suggested that being a non-fitter across different neurological impairments may be underpinned by a common mechanism, however, this commonality could also arise via associations with overall stroke severity. Neurophysiological and neuroimaging biomarkers have not yet been investigated in the context of fitters and non-fitters for measures other than the FM-UE, and is a potential future research option for investigating the recovery of other neurological functions. A recent study dismissed their findings of proportional recovery in the FM-LE due to heteroscedasticity of residuals and other factors;^17^ we discuss this article in the Supplemental Material.

## Problem 1: Mathematical Coupling

**Figure 1.**
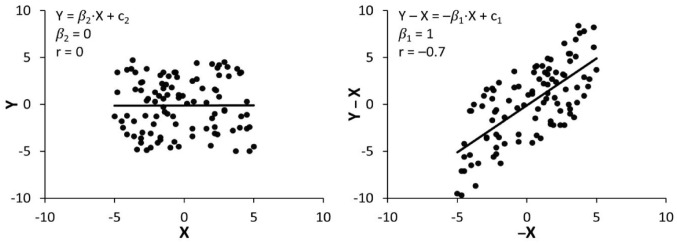
Uncorrelated *X* and *Y* (left) corresponds to correlated *X* and *Y* – *X* (right). *Note. X*, baseline score; *Y*, outcome score; *Y* – *X*, recovery; *β*_1_, proportional recovery slope; *β*_2_, baseline-outcome regression slope; *c*_1_, proportional recovery intercept; *c*_2_, baseline-outcome regression intercept; *r*, correlation coefficient.

Critics have highlighted several supposed confounds of proportional recovery, with some arguing that proportional recovery is spurious, and not valid for modeling post-stroke recovery. One such confound is a statistical concept known as mathematical coupling, which proposes that correlating a variable with a change score containing that same variable is confounded.^18^ Mathematical coupling is commonly referenced in proportional recovery literature,^4,19,20^ featuring prominently in the critiques by Hawe et al,^21^ Lohse et al,^22^ and Bowman et al,^23^ and has also sparked concern in other research fields.^24-30^ For stroke recovery, the canonical mathematical coupling argument suggests that since recovery is equal to outcome minus the baseline score, correlating baseline scores with recovery is confounded because the baseline score appears on both sides of the equation, and thus correlates with itself.^21-23^ This confound is often demonstrated by showing that for random uncorrelated variables *X* and *Y, X* will be correlated with *Y* – *X* with a slope of −1 and a correlation coefficient of roughly −0.71 (Figure 1).^18,22,31^ If a correlation between *X* and *Y* – *X*, hereafter referred to as *r*(*X,Y* – *X*), can arise when there is no correlation between *X* and *Y*, hereafter referred to as *r*(*X,Y*), then proportional recovery could similarly arise with no underlying relationship between baseline and outcome scores, throwing empirical findings of proportional recovery into question. Lastly, the concept of mathematical coupling also applies to the measurement error in *X*. If the measurement error in *X* and *Y* are represented by *ε_X_* and *ε_Y_*, respectively, correlating *X* with *Y* – *X* is said to be amplified by *ε_X_* being present in both variables.^20,28^

### Random Recovery Simulations

**Figure 2.**
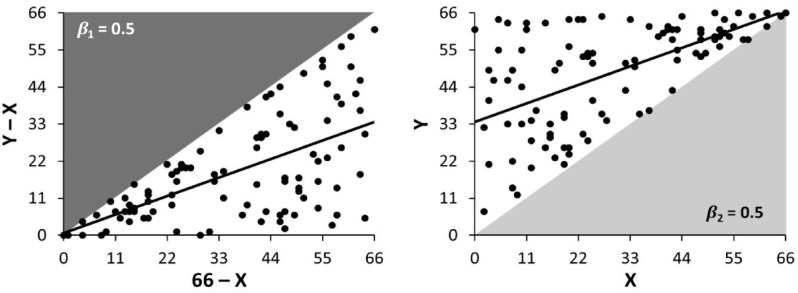
Constrained random simulation results in proportional recovery (left) and baseline-outcome correlation (right). *Note. X*, baseline score; *Y*, outcome score; *Y* – *X*, recovery; *β*_1_, proportional recovery slope; *β*_2_, baseline-outcome regression slope; dark gray area, hard ceiling effect; light gray area, hard floor effect.

Hawe et al^21^ and Lohse et al^22^ argued that due to mathematical coupling, spurious proportional recovery arises even when recovery is “random.” Simulating “random” baseline and outcome FM-UE scores under the constraints that patient scores do not get worse or exceed 66, results in a proportional recovery slope of 50% (Figure 2, left). Since “random” recovery can appear like proportional recovery, empirical findings of proportional recovery could arise from this confound, rather than being underpinned by a true proportional recovery relationship.

### Inflated *R*^2^

Furthermore, it is suggested that strong proportional recovery correlations are misleading, because even if baselines can accurately predict recovery, this does not necessarily mean we can use baselines or predicted recovery to predict outcomes with the same accuracy.^4,21^ Hope et al^4^ showed that when baselines are correlated with recovery but not outcomes, predicted recovery correlates with actual recovery, but predicted outcomes, calculated by summing baseline scores and predicted recovery, do not correlate with actual outcomes. Hawe et al^21^ and Bonkhoff et al^32^ demonstrated that for existing proportional recovery data, baseline scores more strongly correlate with recovery than outcomes. When baselines correlate better with recovery than outcomes, it is argued that the former correlation is spurious, as the high *R*^2^ value gives a false impression that baselines can also be used to predict outcomes.

## Rebuttal: Mathematical Coupling

**Figure 3.**
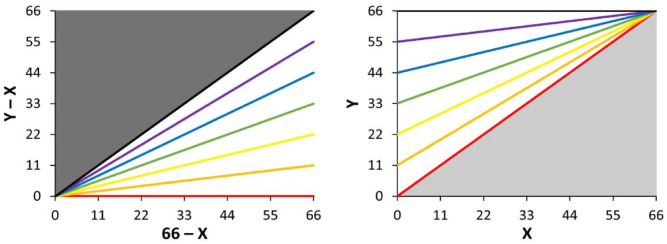
Pairwise regression lines for proportional recovery (left) and baseline-outcome regression (right). *Note. X*, baseline score; *Y*, outcome score; *Y* – *X*, recovery; *β*_1_, proportional recovery slope; *β*_2_, baseline-outcome regression slope; black lines, *β*_1_ = 1, *β*_2_ = 0; purple lines, *β*_1_ = .83, *β*_2_ = .17; blue lines, *β*_1_ = .67, *β*_2_ = .33; green lines, *β*_1_ = .5, *β*_2_ = .5; yellow lines, *β*_1_ = .33, *β*_2_ = .67; orange lines, *β*_1_ = .17, *β*_2_ = .83; red lines, *β*_1_ = 0, *β*_2_ = 1; dark gray area, hard ceiling effect; light gray area, hard floor effect.

First, we shall address mathematical coupling of the true measurement value, disregarding measurement error. Proportional recovery (equation (1)) and baseline-outcome regression (equation (2)) are geometric transformations of each other (Figure 3), that model the same fundamental relationship. When equivalating equations (1) and (2) (equation (3)), it follows that the slopes of proportional recovery and baseline-outcome regression always sum to 1 (equation (4)).

(1) $$Y-X=\beta_{1}\cdot (\max\mathrm{score}-X)+c_{1}$$

(2) $$Y=\beta_{2}\cdot X+c_{2}$$

(3) $$\begin{array}{c} \beta_{1}\cdot \left(\max\mathrm{score}-X\right)+c_{1}=\beta_{2}\cdot X+c_{2}-X\\ \beta_{1}\cdot X-\beta_{1}\cdot \max\mathrm{score}-c_{1}=(1-\beta_{2})\cdot X-c_{2} \end{array}$$

(4) $$\begin{array}{c} \beta_{1}=1-\beta_{2}\\ \beta_{2}=1-\beta_{1} \end{array}$$

$$c_{2}=\beta_{1}\cdot \mathrm{ceiling}+c_{1}$$

**Figure 4.**
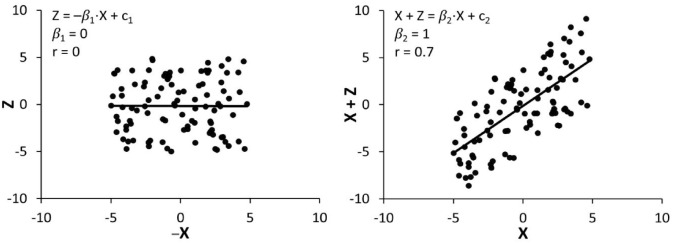
Uncorrelated *X* and *Z* (left) corresponds to correlated *X* and *X* + *Z* (right). *Note. X*, baseline score; *Z*, recovery; *X* + *Z*, outcome score; *β*_1_, proportional recovery slope; *β*_2_, baseline-outcome regression slope; *c*_1_, proportional recovery intercept; *c*_2_, baseline-outcome regression intercept; *r*, correlation coefficient.

Next, *R*^2^ is equal to 1 minus the ratio of the residual sum of squares to the total sum of squares. Since proportional recovery and baseline-outcome regression have identical residuals, the difference between their *R*^2^ (and by extension, *r*) depends on their total sum of squares, which in turn depends on their regression slope. In terms of magnitude, when *β*_1_ = *β*_2_ = 0.5, *r*(*X,Y*) will equal *r*(*X,Y* – *X*), while for *β*_1_ < .5<*β*_2_, *r*(*X,Y*) will exceed *r*(*X,Y* – *X*), and for *β*_2_ < .5 < *β*_1_, *r*(*X,Y* – *X*) will exceed *r*(*X,Y*). The issue with showing that for uncorrelated *X* and *Y, X* correlates with *Y* – *X* (Figure 1),^18,22,31^ is that it refers to the specific situation where *β*_1_ = 1, *β*_2_ = 0 (Figure 3, black lines). This is only one of many different possible combinations of *β*_1_ and *β*_2_, for example, the scenario *β*_1_ = 0, *β*_2_ = 1 (Figure 3, red lines) describes that for uncorrelated *X* and *Y* – *X, X* correlates with *Y* (Figure 4). In stroke recovery, it is rarely the case that baselines and outcomes have zero correlation. For any *β*_1_ < .5 < *β*_2_, baselines will correlate more strongly with outcomes than recovery, and the proposition that proportional recovery is amplified by coupling no longer holds. Essentially, the common simulation of mathematical coupling only shows a narrow glimpse of what is a more nuanced relationship between the slopes and correlation coefficients of baselines, outcomes, and recovery.

The correlation *r*(*X,Y* – *X*) is said to be mathematically coupled because *X* is present in both variables. However, a term appearing on both sides of the equation is insufficient evidence that the correlation is confounded. First, *r*(*X,Y* – *X*) could equal zero, so mathematical coupling is clearly insufficient to result in a spurious correlation. Secondly, if we define a new variable *Z* = *Y* – *X*, we can express *r*(*X,Y* – *X*) as *r*(*X,Z*), which no longer appears to have a self-correlating component. Of course, it could be argued that *Z* indirectly contains *X*, which is technically true, but we could similarly argue that *Y* indirectly contains *X* since *Y* = *X* + *Z*. The correlation *r*(*X,Y*) can be expressed as *r*(*X,X* + *Z*), which now appears to contain a self-correlating *X* component, and would be considered mathematically coupled. Essentially, any correlation that appears to be coupled can be written in a form where it is not coupled, and any non-coupled correlation can be written in a form where it is coupled. Naturally, critiques of proportional recovery focus on the notation *r*(*X,Y* – *X*), which is the more intuitive notation for stroke recovery since baseline and outcome scores are empirically measured while change scores are calculated. However, other than determining the source of measurement error, it should not really matter which variables are empirically measured or calculated, since coupling is a mathematical phenomenon. Disregarding measurement error, *r*(*X,Y* – *X*) and *r*(*X,Z*) are the same correlation, so it does not make sense that the former be confounded while the latter not. Since coupling can be produced, or eliminated from any correlation via changes to its notation, which importantly does not affect the value of *r*, the apparent presence or absence of mathematical coupling is ultimately inconsequential. In summary, mathematical coupling of the true measurement value is not a true statistical confound, as it is simply a notational construct which makes no difference to the strength of a correlation, and is not a sufficient condition to render a correlation confounded.

### Rebuttal: Random Recovery Simulations

The random recovery argument relies on the observation that a proportional recovery relationship arises when recovery is “random.” However, the simulations in Hawe et al^21^ are not truly random, as they impose a hard ceiling of 66 points for all scores, and assume recovery is positive. While these constraints are sensible, it means that recovery cannot exceed baseline impairment, making recovery only pseudo-random as it is no longer independent from baseline score. The resulting proportional recovery relationship is only natural since the variables are partially dependent. These simulations merely demonstrate that proportional recovery arises when simulating pseudo-random data under conditions that make baselines and recovery dependent, which should be a given, and to us does not suggest that proportional recovery is confounded. If these constraints were not sensible, then the corresponding proportional recovery relationship could be considered artifactual. However, these constraints represent our fundamental understanding of post-stroke recovery, that people generally get better after stroke, and do not recover more than what they lost. Therefore, the associated proportional recovery relationship should be valid.

The emergence of proportional recovery in these simulations is typically attributed to mathematical coupling,^21,22^ but is actually due to the hard ceiling effect. If baseline scores and recoveries are randomly generated with no hard ceiling effect, proportional recovery does not arise (Figure 4, left).

### Rebuttal: Inflated *R* ^2^

First, the most obvious rebuttal to the argument that mathematically coupled correlations have inflated *R*^2^ estimates is the demonstrable fact that, disregarding measurement error, having the same term appear on both sides of the equation makes no difference to the *R*^2^ of that correlation, that is *r*(*X,Y* – *X*) = *r*(*X,Z*), where *Z* = *Y* – *X*. As previously mentioned, whether variables are empirically measured or calculated is only relevant for coupling of the measurement error, which is discussed later.

The argument that strong *r*(*X,Y* – *X*) are inflated when *r*(*X,Y*) is weak relies on the assumption that if baselines can predict recovery, they should also predict outcomes with the same accuracy.^4,21^ This assumption holds true if accuracy is measured using prediction residuals or related metrics, which are identical for proportional recovery and baseline-outcome regression. However, in terms of *R*^2^ or *r*, the correlation between predicted recovery and actual recovery (equivalent to *r*(*X,Y* – *X*)) can differ from the correlation between predicted recovery plus baselines, and actual outcomes (equivalent to *r*(*X,Y*)), because as we have previously explained, *r*(*X,Y* – *X*) differs from *r*(*X,Y*) based on their regression slopes. Thus, the inflated *R*^2^ argument does not hold, since its premise that *r*(*X,Y* – *X*) and *r*(*X,Y*) are similar in strength is demonstrably false.

Lastly, we accept that if 2 correlations have identical residuals but discrepant *R*^2^, the larger *R*^2^ estimate could be considered inflated or misleading, but only if one believes that higher *R*^2^ means smaller residuals, which is not always the case. Thus, the problem of inflated *R*^2^ is due to false perception, not the estimate itself. Nevertheless, it is possible that researchers could “hack” their correlations by selectively reporting the stronger *R*^2^ value, and this could mislead readers unfamiliar with this discourse. Researchers should take care when interpreting *R*^2^ statistics of proportional recovery and baseline-outcome regression, and consider alternative measures of model performance like mean average error or mean squared error, particularly for evaluating prediction accuracy.

#### Mathematical Coupling of the Measurement Error

Lastly, we must examine mathematical coupling of the measurement error. Consider that empirical scores are equal to true scores plus measurement error (*X*_emp_ = *X*_true_ + *ε_X_*, and *Y*_emp_ = *Y*_true_ + *ε_Y_*), thus calculated recovery is equal to *Y*_emp_ – *X*_emp_, or *Y*_true_+ *ε_Y_* – *X*_true_ – *ε_X_*. Correlating empirical baseline scores with calculated recovery now encounters mathematical coupling of the error term *ε_X_*. Using our previous logic, we could “hide” *ε_X_* by using alternative notation, but this would not escape the fact that both *r*_emp_(*X,Y* – *X*) and *r*_emp_(*X,Z*) are correlations between empirical scores. These empirical correlations can differ from the true correlations *r*_true_(*X,Y*) and *r*_true_(*X,Y* – *X*), where the discrepancy depends on *ε_X_* and *ε_Y_*.

**Figure 5.**
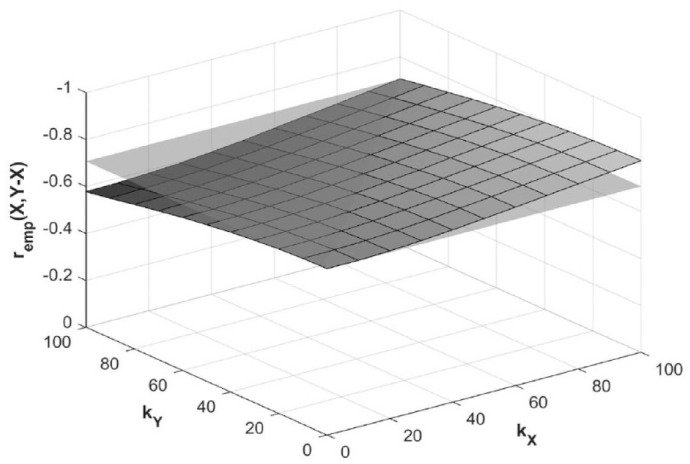
Effect of error magnitude on empirical *r*_emp_(*X,Y* – *X*) for canonical mathematical coupling scenario. *Note. X*, baseline score; *Y*, outcome score; *Y* – *X*, recovery; *k_X_*, error magnitude in *X; k_Y_*, error magnitude in *Y; r*_emp_(*X,Y* – *X*), empirical correlation coefficient between baseline scores and recovery; gray plane, true correlation coefficient *r*_true_(*X,Y* – X) = –0.71.

We investigated the effect of *ε_X_* and *ε_Y_* on the correlations *r*_emp_(*X,Y*) and *r*_emp_(*X,Y* – *X*), compared to the true correlations *r*_true_(*X,Y*) and *r*_true_(*X,Y* – *X*). Assuming *ε_X_* and *ε_Y_* are independent, we generated random *X*_true_ (range 0–100), *ε_X_* (range 0–*k_X_*), and *ε_Y_* (range 0–*k_Y_*), and varied the value of *k_X_* and *k_Y_* for 3 different functions of *Y*_true_. Full methods are available in the Supplemental Material. For the canonical example of mathematical coupling where *r*_true_(*X,Y*) is 0 and *r*_true_(*X,Y* – *X*) is −0.71, increasing *k_X_* will amplify *r*_emp_(*X,Y* – *X*), to about −0.74 when *k_X_* = 50, and −0.82 when *k_X_* = 100. Contrarily, increasing *k_Y_* attenuates *r*_emp_(*X,Y* – *X*), to the extent that any *ε_X_*-based amplification of *r*_emp_(*X,Y* – *X*) is completely offset when *k_Y_* = *k_X_* (Figure 5).

**Figure 6.**
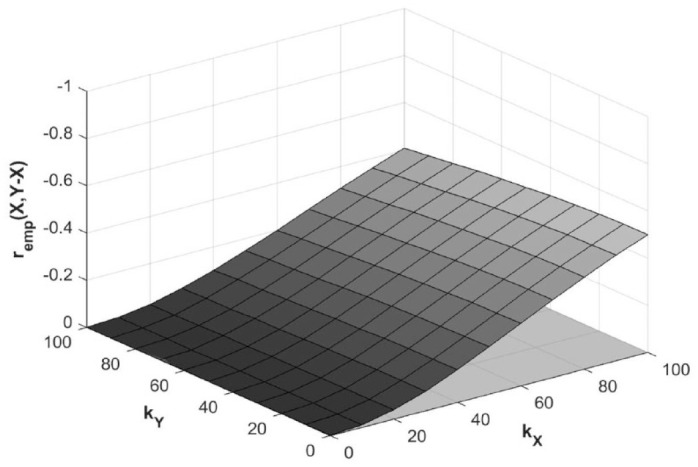
Effect of error magnitude on empirical *r*_emp_(*X,Y* – *X*) for random recovery scenario. *Note. X*, baseline score; *Y*, outcome score; *Y* – *X*, recovery; *k_X_*, error magnitude in *X; k_Y_*, error magnitude in *Y; r*_emp_(*X,Y* – *X*), empirical correlation coefficient between baseline scores and recovery; gray plane, true correlation coefficient *r*_true_(*X,Y* – *X*) = 0.

When true recovery is random, that is, *r*_true_(*X,Y* – *X*) is 0, and *r*_true_(*X,Y*) is −0.71, increasing *k_X_* will spuriously amplify *r*_emp_(*X,Y* – *X*) to about −0.2 when *k_X_* = 50, and about −0.5 when *k_X_* = 100. Increasing *k_Y_* will attenuate spurious *r*_emp_(*X,Y* – *X*), but only by about 8% at *k_Y_* = 50 and 25% at *k_Y_* = 100 (Figure 6).

**Figure 7.**
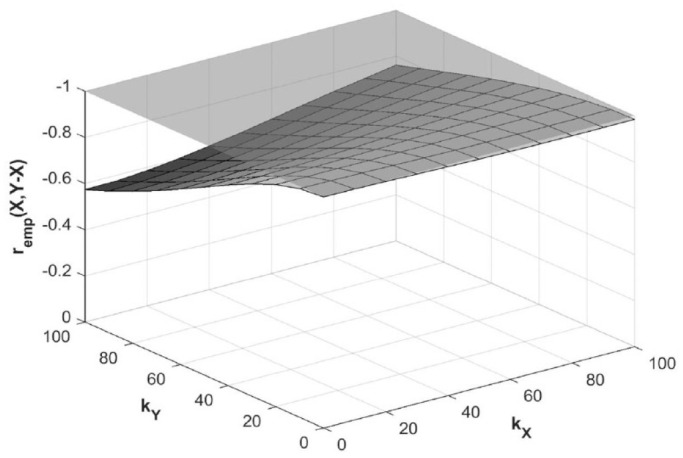
Effect of error magnitude on empirical *r*_emp_(*X,Y* – *X*) for true proportional recovery scenario. *Note. X*, baseline score; *Y*, outcome score; *Y* – *X*, recovery; *k_X_*, error magnitude in *X; k_Y_*, error magnitude in *Y; r*_emp_(*X,Y* – *X*), empirical correlation coefficient between baseline scores and recovery; gray plane, true correlation coefficient *r*_true_(*X,Y* – *X*) = –1.

Lastly, if the true relationship is 70% proportional recovery, that is *r*_true_(*X,Y* – *X*) is −1, and *r*_true_(*X,Y*) is 1, increasing *k_Y_* will attenuate *r*_emp_(*X,Y* – *X*). Increasing *k_X_* will amplify *r*_emp_(*X,Y* – *X*) to a smaller extent, offsetting *ε_Y_*-based attenuation by about 20% when *k_X_* = 50, and 50% when *k_X_* = 100. This ultimately reduces, rather than increases, the disparity between *r*_emp_(*X,Y* – *X*) and *r*_true_(*X,Y* – *X*; Figure 7).

Since *r*(*X,Y*) is not affected by error coupling, increasing *k_X_* and *k_Y_* only attenuate *r*_emp_(*X,Y*) relative to *r*_true_(*X,Y*) since adding error dilutes the relationship (Supplemental Figures 1, 2, and 3).

In summary, mathematical coupling of the measurement error can amplify empirical proportional recovery correlation coefficients, but the effect is relatively small unless the variance in ε_X_ is large. This confound is unlikely to spuriously produce statistically significant proportional recovery relationships out of nothing, since real data should have lower measurement error variance than our simulations, but could push near-significant proportional recovery estimates into statistical significance. However, most proportional recovery relationships in the existing literature are well past the threshold of statistical significance,^1,6,8,9,13^ and should remain valid.

## Problem 2: Ceiling Effects

Another common criticism of proportional recovery is about ceiling effects, particularly for the FM-UE. For clarity, we shall refer to absolute score ceilings, such as the maximum score of 66 in the FM-UE, as the hard ceiling effect, and reduced score variability as scores approach the ceiling as the soft ceiling effect.

### Hard Ceiling Effect

Clinical assessments with score ceilings cannot measure performance above their maximum score and thus have a limited measurement range. The FM-UE is commonly criticized for its hard ceiling effect, as patients who achieve the maximum score of 66 may still have upper limb motor impairments that are not captured by the scale.^33^ Due to the hard ceiling effect, all outcome scores at or hypothetically above the score ceiling will be truncated at ceiling, where individually, they are equal to 100% recovery from impairment, and thus strengthen the proportional recovery relationship. When simulating random baseline FM-UE scores, constant recovery of 33 points, and a hard ceiling of 66, Hope et al^4^ observed a proportional recovery relationship, which persisted even when randomly shuffling the outcome scores. The hard ceiling effect is stronger for patients with mild baseline impairments, since the likelihood of reaching ceiling at follow-up increases with higher baseline scores.^34^

Similarly, Bonkhoff et al^32^ showed that proportional to spared and constant recovery functions can appear like proportional recovery if a hard ceiling effect is imposed. It is said that empirical findings of proportional recovery may be unreliable, since different recovery functions operating under a hard ceiling effect can appear like proportional recovery.

### Soft Ceiling Effect

Since generally, people get better after stroke, and do not recover more than what they lose, people with low baseline scores have more possible outcome scores than those with high baseline scores. For example, someone with a baseline FM-UE of 10 has 57 possible outcome scores, while someone with a baseline FM-UE of 60 only has 7 possible outcome scores, assuming neither person gets worse. This means that score variability decreases as scores approach ceiling. Since patients generally get better over time, outcome scores are less variable than baseline scores, a phenomenon known as compression toward ceiling. In addition, any truncation of scores by the hard ceiling effect will also contribute to reduced score variability.

Mathematical proofs have shown that *r*(*X,Y* – *X*) is a function of *r*(*X,Y*) and the ratio of the outcome standard deviation to baseline standard deviation (variability ratio), often visualized using a 3-dimensional surface plot.^4,32^ When the variability ratio is low, *r*(*X,Y* – *X*) will always be negative, regardless of the strength of *r*(*X,Y*).^4^ Consequently, several articles suggest that a low variability ratio causes spurious proportional recovery to arise, and since compression toward ceiling is a common property of real stroke recovery data, that proportional recovery is inevitable.^4,21,32^

## Rebuttal: Ceiling Effects

### Rebuttal: Hard Ceiling Effect

It is true that truncation of outcome scores at the hard ceiling contributes to a stronger proportional recovery relationship. However, the ability to perform outside the measurable range of a given scale does not invalidate findings of proportional recovery in that scale. Consider the patients who reach ceiling in the Hope et al constant recovery simulation, that is, those with baseline FM-UE ≥ 33, constant recovery of 33 points, and a full score of 66 at follow-up. Even if theoretical recovery beyond the score ceiling of 66 were possible, it would hold true that these patients recover 100% of their baseline impairment within the measurement range of the FM-UE. The hard ceiling effect introduces the caveat that this relationship may not hold for theoretical FM-UE scores above 66, however, this is only appropriate since a model derived from FM-UE data should not be expected to characterize recovery dynamics for impairments that the FM-UE does not measure.

As suggested by Bonkhoff et al,^32^ the hard ceiling effect can make different recovery patterns appear like proportional recovery. However, the likelihood that an outcome score reaches ceiling under a proportional to spared or constant recovery pattern is greatest for patients with mild baseline impairments, which is precisely the subset of patients for whom these recovery patterns are unrealistic. This is because patients generally do not recover to better than their pre-stroke performance, but proportional to spared or constant recovery functions suggest that recovery remains positive even as impairment approaches zero. Mechanistically, it is only natural that recovery operates within the bounds of the post-stroke impairment, and that there be a biological ceiling representing the maximum performance achievable via spontaneous recovery, regardless of whether this is accurately captured by the ceiling of the measurement scale. This is consistent with proportional recovery, but not proportional to spared or constant recovery functions. While other recovery functions could possibly apply to patients with more severe baseline impairments,^17^ this may be attributable to the fitter/non-fitter dichotomy, which is better explained with neurophysiological or neuroimaging biomarkers. Even if proportional to spared or constant recovery patterns were feasible, they would appear as a bimodal relationship, since ceiled datapoints would be equal to 100% recovery from impairment, and non-ceiled datapoints would represent the given recovery function. However, Goldsmith et al^35^ found that recovery in fitters was best modeled by linear, rather than nonlinear functions.

Furthermore, analyzing outcomes instead of recovery does not overcome the limitations of a scale with hard ceiling effects. For example, if recovery is constant, better baselines should correspond to better outcomes, but in the above simulation it would appear that all patients with baseline FM-UE scores between 33 and 66 have the same outcome. Similarly, generating random baselines and either outcomes or recovery with the same hard ceiling constraints as Hawe et al^21^ not only results in a proportional recovery slope of 0.5, but also a baseline-outcome regression slope of 0.5 (Figure 2, right). While we agree that the FM-UE is affected by the hard ceiling effect, this is a limitation of the scale, rather than proportional recovery. Similarly, other issues with the FM-UE, such as its nonlinearity, rounding error, and in equivalence of test items,^20,22,32,33^ are related to the FM-UE rather than the statistical method used to analyze it.

### Rebuttal: Soft Ceiling Effect

Proportional recovery is said to be confounded, because a low variability ratio inevitably causes strong proportional recovery. However, while proportional recovery, baseline-outcome regression, and the variability ratio are intrinsically linked,^4,32^ this does not entail 1-way causality. Technically, as the variability ratio decreases, proportional recovery becomes stronger, but the same could be said that as proportional recovery becomes stronger, the variability ratio decreases. Thus, the premise that a low variability ratio causes proportional recovery is a 1-sided interpretation of the fact that these phenomena are reciprocally associated.

It is true that when the variability ratio is low, proportional recovery will inevitably occur, regardless of the relationship between baselines and outcomes; for example, proportional recovery will persist even when outcome scores are shuffled.^4^ However, the premise that if we observe A, we must inevitably observe B, does not mean that observation B is confounded. Furthermore, proportional recovery should be able to exist regardless of whether baselines correlate with outcomes or not. The surface plot of the relationship between proportional recovery, baseline-outcome regression, and variability ratio, clearly shows that for negative *r*(*X,Y* – *X*), any *r*(*X,Y*) is possible.^4,32^ Suggestions that proportional recovery should be accompanied with a correlation between baselines and outcomes, ventures back into mathematical coupling territory and the misconception that *r*(*X,Y*) approximates *r*(*X,Y* – *X*).

Some critiques of proportional recovery suggest that compression toward ceiling is an unwanted confound that masks the true variability in recovery.^23,32^ Instead, we suggest that compression toward ceiling is a valid representation of our understanding of post-stroke recovery. Since generally, people get better after stroke and do not exceed their pre-stroke performance, the possible outcomes for someone recovering from stroke should lie between their pre-stroke performance and their baseline post-stroke score. These recovery properties, akin to the constraints imposed in “random” recovery simulations,^21,22^ mean that outcome score variance must be lower than baseline score variance, thus resulting in compression toward ceiling. Furthermore, most recovery occurs early after stroke, after which performance becomes relatively stable. This means that over time, recovery rates, and thus score variability, should also decrease. In summary, compression toward ceiling is a valid representation of our current understanding of post-stroke recovery, and so the corresponding proportional recovery relationship should also be valid since they are related phenomena. We therefore suggest that compression toward ceiling and proportional recovery are not unwanted confounds, but rather inherent properties of post-stroke recovery dynamics.

## Closing Remarks

Proportional recovery is a valid group-level model for describing spontaneous recovery from post-stroke impairment, and its supposed confounds, namely mathematical coupling and ceiling effects, have disputable foundations. Further discussion relating to these confounds is available in the Supplemental Material. The exception is mathematical coupling of the measurement error, which can amplify the proportional recovery correlation coefficient; however, this makes little difference unless the variance in baseline score measurement error is high. Notably, various other supposed confounds and limitations of proportional recovery have been mentioned in the literature, such as nonlinearity, heteroscedasticity, and the classification methods for fitters and non-fitters. These concerns are discussed in the Supplemental Material.

Proportional recovery and baseline-outcome regression are geometric transformations of each other, and can be thought of as 2 different, but fundamentally related approaches for analyzing longitudinal data. Testing for proportional recovery instead of baseline-outcome regression may be useful when the variable of interest is recovery rather than outcome, although researchers should consider that proportional recovery estimates may be slightly amplified by mathematical coupling of the measurement error. However, since correlations between baselines and outcomes after stroke are relatively commonplace, similarly, correlations between baselines and recovery should be unsurprising. Just like baseline-outcome regression, proportional recovery as a general statistical concept is not very interesting, as it does not teach us anything particularly new about recovery. Consequently, we suggest that researchers need not demonstrate that proportional recovery exists in new stroke populations or different measurement scales for the sake of generalizability.

Compression toward ceiling and proportional recovery are inherently related to and consistent with our understanding of recovery dynamics, so in a way, proportional recovery could be said to represent spontaneous biological recovery after stroke as previously suggested.^1-3^ However, this does not necessarily mean a linear relationship or a specific recovery proportionality applies universally across all post-stroke impairments; the inevitability of a correlation between baseline score and recovery does not entail that this linear relationship is always the best model for post-stroke recovery, and the recovery proportionality may differ across clinical scales, since the amount of recovery that occurs within a scale also depends on what that scale measures. For instance, while recovery in the FM-UE appears to be best modeled by proportional recovery,^35^ recovery in other measurement scales or neurological domains could have different recovery proportionalities, or have a non-linear relationship with baseline score.

Whether through proportional recovery or baseline-outcome regression, baseline scores should be the starting point for investigating recovery after stroke. However, baseline scores alone cannot explain some inter-individual variability in post-stroke recovery, such as the fitter/non-fitter dichotomy, which is better explained by neurophysiological or neuroimaging biomarkers. We suggest using multivariable approaches, leveraging biomarkers, or using serial measurements of performance when investigating outcome and recovery after stroke. In doing so, it is crucial to include baseline impairment as a potential predictor or covariate, since correlations between baselines and recovery and/or outcome are inevitable. Only then can we better understand the factors that determine post-stroke recovery and outcome.

## Supplemental Material

sj-docx-1-nnr-10.1177_15459683231177598 – Supplemental material for Proportional Recovery After Stroke: Addressing Concerns Regarding Mathematical Coupling and Ceiling Effects Click here for additional data file. Supplemental material, sj-docx-1-nnr-10.1177_15459683231177598 for Proportional Recovery After Stroke: Addressing Concerns Regarding Mathematical Coupling and Ceiling Effects by Benjamin Chong, Alan Wang and Cathy M. Stinear in Neurorehabilitation and Neural Repair
