# Supplementary material for: Proportional Recovery After Stroke: Addressing Concerns Regarding Mathematical Coupling and Ceiling Effects
Source: Neurorehabil Neural Repair. 2023 Jun 2;37(7):488–98. doi: 10.1177/15459683231177598 (PMC10350731; doi:10.1177/15459683231177598)
Supplement: sj-docx-1-nnr-10.1177_15459683231177598 – Supplemental material for Proportional Recovery After Stroke: Addressing Concerns Regarding Mathematical Coupling and Ceiling Effects [file sj-docx-1-nnr-10.1177_15459683231177598.docx]

**SUPPLEMENTAL MATERIAL**

**for article “Proportional Recovery After Stroke: Addressing Concerns Regarding Mathematical Coupling and Ceiling Effects”**

**Supplemental Methods: Mathematical Coupling of the Measurement Error**

To investigate the effect of ε_X_ and ε_Y_ on r_emp_(X,Y) and r_emp_(X,Y–X), compared to r_true_(X,Y) and r_true_(X,Y–X), we simulated random data using the following methods. For each simulation, we generated random X_true_ from 0 to 100, random ε_X_ from 0 to k_X_, and random ε_Y_ from 0 to k_Y_, for all combinations of k_X_ and k_Y_ with possible values of 10, 20, …, 90, 100. For scenario 1, canonical mathematical coupling, Y_true_ was randomly generated from 0 to 100. For scenario 2, random recovery, we randomly generated Z_true_ between 0 and 100, and calculated Y_true_ as X_true_+Z_true_. For scenario 3, 70% proportional recovery, we calculated Y_true_ as X_true_+0.7(100-X_true_). All random number generation was performed for 1000 iterations, with non-integers permitted. For all scenarios, X_emp_ was calculated as X_true_+ε_X_, and Y_emp_ was calculated as Y_true_+ε_Y_. The true correlations r_true_ were calculated using X_true_ and Y_true_, while the empirical correlations were calculated using X_emp_ and Y_emp_.


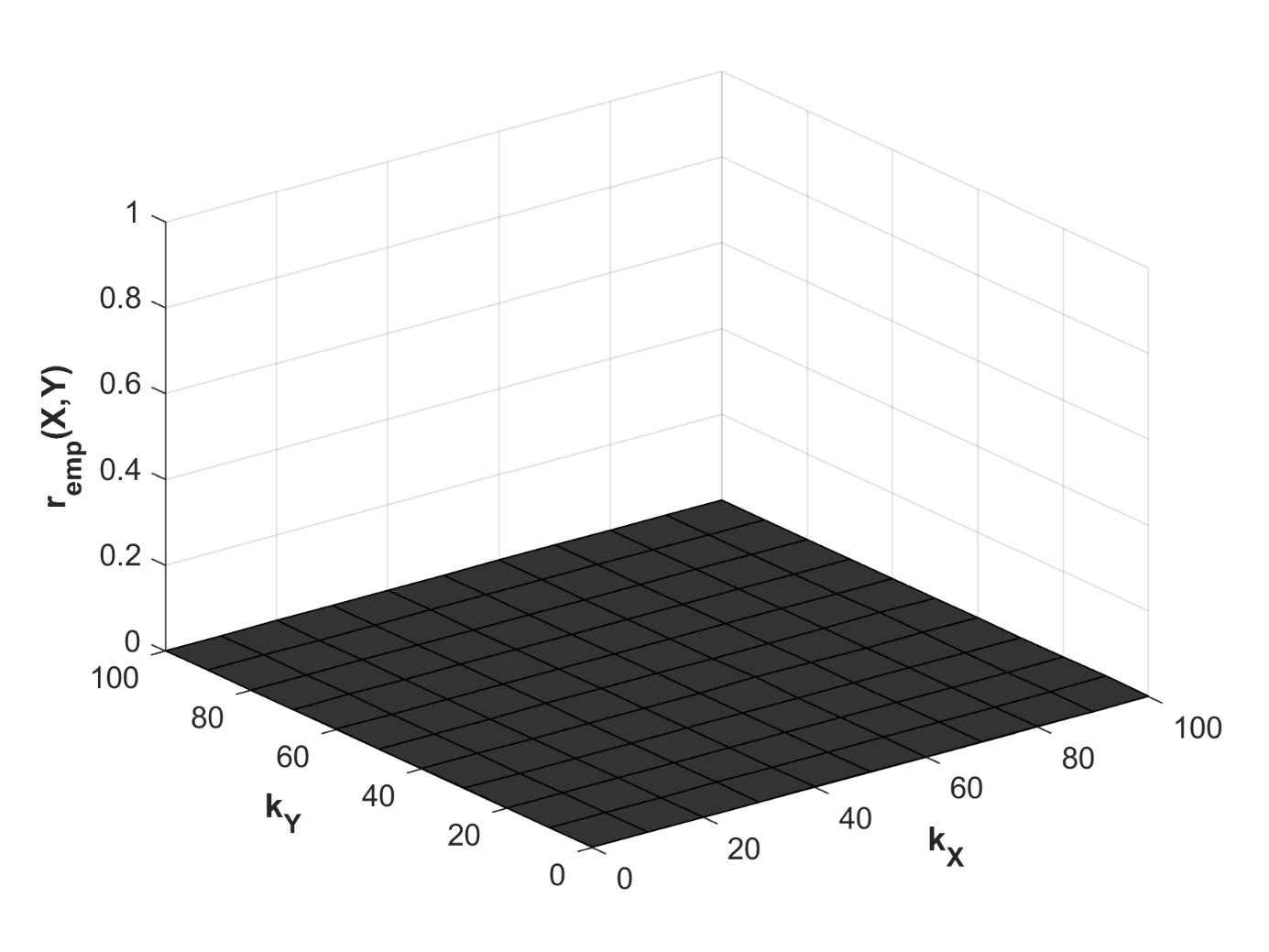


**Supplemental Figure 1**

**Effect of error magnitude on empirical r_emp_(X,Y) for canonical mathematical coupling scenario**

X, baseline score; Y, outcome score; k_X_, error magnitude in X; k_Y_, error magnitude in Y; r_emp_(X,Y), empirical correlation coefficient between baseline scores and outcome scores; true correlation coefficient r_true_(X,Y) = 0 (hidden).


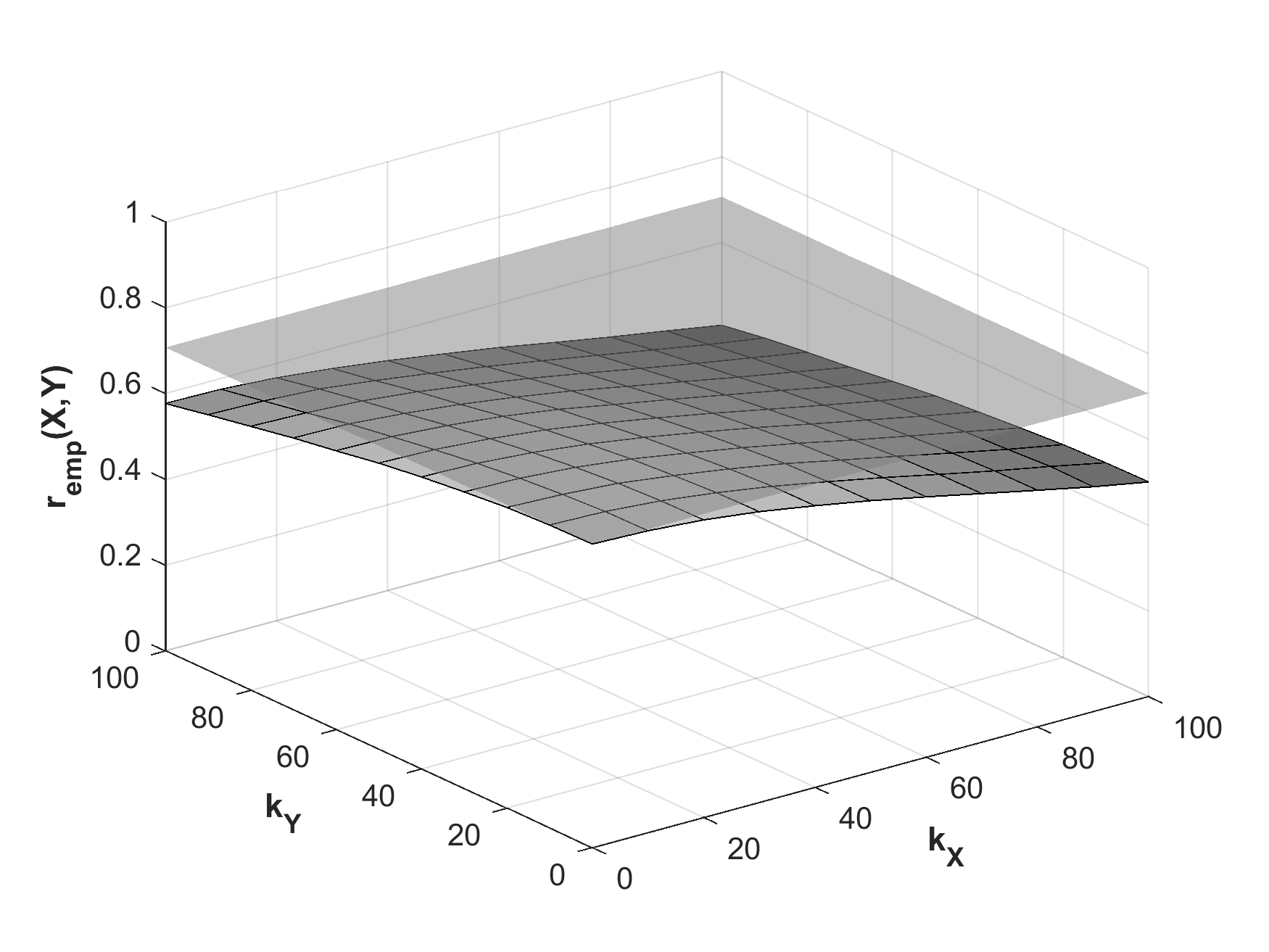


**Supplemental Figure 2**

**Effect of error magnitude on empirical r_emp_(X,Y) for random recovery scenario**

X, baseline score; Y, outcome score; k_X_, error magnitude in X; k_Y_, error magnitude in Y; r_emp_(X,Y), empirical correlation coefficient between baseline scores and outcome scores; grey plane, true correlation coefficient r_true_(X,Y) = 0.71.


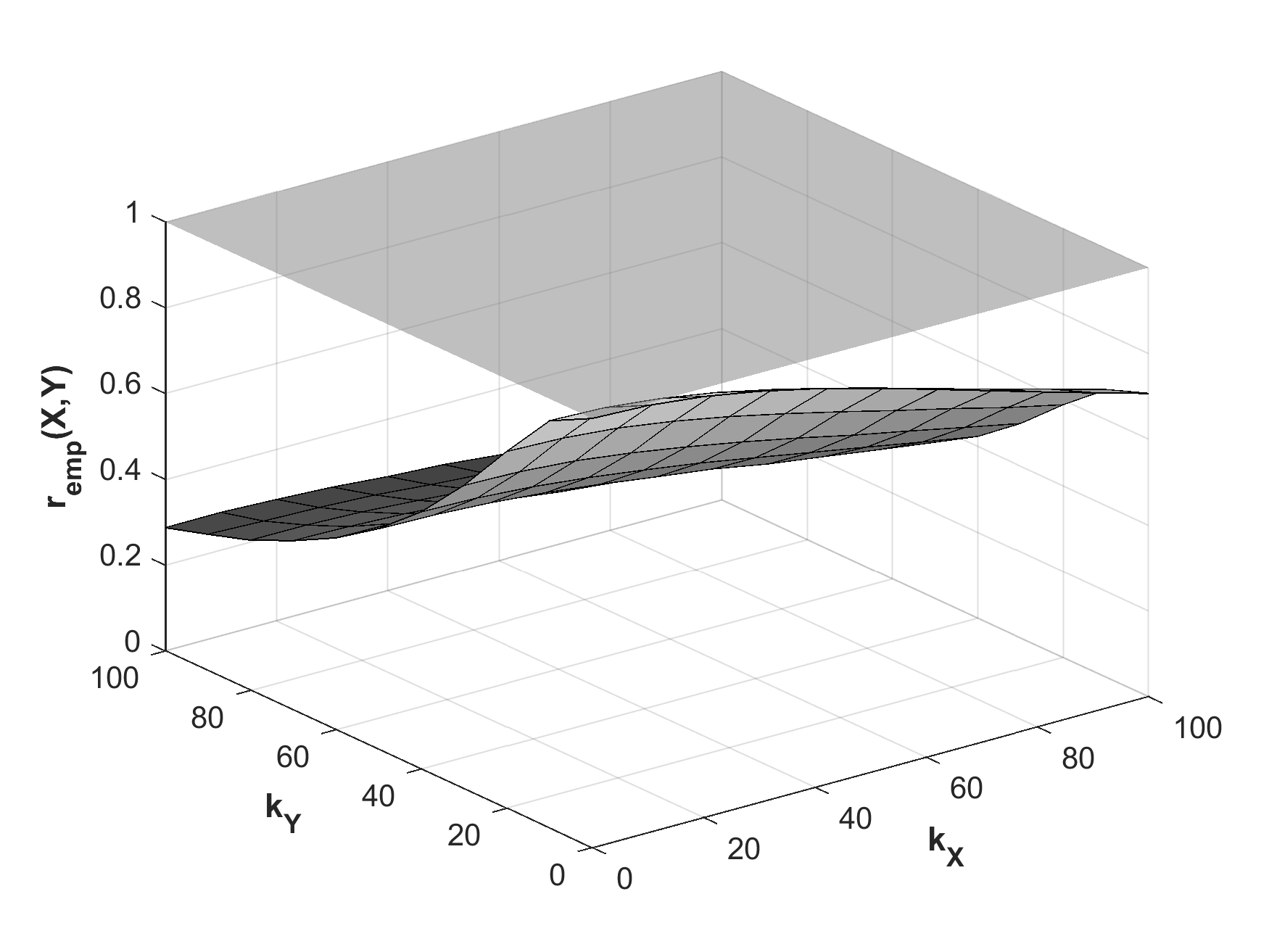


**Supplemental Figure 3**

**Effect of error magnitude on empirical r_emp_(X,Y) for true proportional recovery scenario**

X, baseline score; Y, outcome score; k_X_, error magnitude in X; k_Y_, error magnitude in Y; r_emp_(X,Y), empirical correlation coefficient between baseline scores and outcome scores; grey plane, true correlation coefficient r_true_(X,Y) = 1.

**Additional Evidence Against the Inflated R^2^ Argument**

The argument that a high proportional recovery R^2^ is misleading when it exceeds baseline-outcome regression R^2^ takes the form: if r(X,Y–X) exceeds r(X,Y), then r(X,Y–X) is inflated. Defining Z=Y–X, this argument can also be expressed as: if r(X,Z) exceeds r(X,X+Z), then r(X,Z) is inflated. When the slope of r(X,Z) is greater than 0.5, r(X,Z) will exceed r(X,X+Z), even though they have the same residuals. Since X, Y, and Z could theoretically be anything, these arguments can be extended to any and all linear correlations, not just proportional recovery. Therefore, if the inflated R^2^ argument was true, any and all linear correlations with a slope greater than 0.5 would be inflated, since they have a geometrically related correlation with a lower R^2^ but identical residuals.

**Proportional Recovery in the Purdue Pegboard Test**

In Hawe et al., data from the Purdue Pegboard Test, which has no hard ceiling, was found to exhibit proportional recovery.^1^ The proportional recovery R^2^ for the Purdue Pegboard Test was lower than what was found for the Functional Independence Measure, a scale with hard ceiling effects, so the authors interpreted this as evidence against the validity of proportional recovery. However, the low R^2^ can also be explained by the method used to calculate impairment for the Purdue Pegboard Test. Theoretically, at the individual level, the true extent of impairment caused by a stroke is the difference between a patient’s pre-stroke and post-stroke performance, however pre-stroke performance is not practically measurable. Calculating impairment is simple for scales like the FM-UE, which has a maximum score of 66 that can be used as a proxy for pre-stroke performance. The Purdue Pegboard Test has no such reference score, so Hawe et al. defined pre-stroke performance as a fixed score of 17 points. However, the true pre-stroke performance on the Purdue Pegboard Test in their sample is likely to vary between individuals, rather than be a fixed score. Using a fixed score of 17 points to calculate impairment fails to account for this variability, and each individual impairment score will theoretically be contaminated by an error equal to the difference between 17 and the individual’s true pre-stroke performance. This would have contributed towards the lower proportional recovery R^2^ for the Purdue Pegboard Test, and we suggest that the positive finding of proportional recovery in the Purdue Pegboard Test supports the concept of proportional recovery after stroke.

**Correction to Equations in Article by Lohse et al., “Statistical Considerations for Drawing Conclusions About Recovery”**

For the sake of accuracy, we note that two of the equations reported in Lohse et al., used in support of their arguments against proportional recovery, are incorrect.^2^ In their Equation 2A, change scores are said to be represented by the equation: Y–X=c_1_+error, while their Equation 2B states that outcome scores are equal to Y=X+c_1_+error (notation modified for consistency with the present article). Both these equations are missing the recovery term *β*_1_(–X), and incorrectly suggest that recovery can be modeled purely by a constant plus error. However, the arguments drawn from these equations in Lohse et al. have limited relevance to the main ideas of mathematical coupling.

**Nonlinearity and Variations in Slope**

In their critique of proportional recovery, Hawe and colleagues demonstrated that the recovery proportionalities for individual patients have large inter-individual variability.^1^ However, calculating the recovery proportionality for each observation fundamentally differs from linear regression modelling at the group level. Individual recovery proportionalities calculated by the former approach can be highly skewed by random variation, especially for data points close to the origin.

Two studies have demonstrated that local slope variations for proportional recovery can exist for certain windows of FM-UE scores.^3,4^ Lee et al., found that the proportional recovery slope differed between patients with higher and lower baseline FM-UE.^3^ The authors suggested that the relationship between baseline FM-UE and recovery is nonlinear, and that a linear model like proportional recovery is unsuitable for modelling this relationship. However in their study, 'lower' baseline FM-UE spanned from 10 to 60 points, while 'higher' baseline FM-UE spanned from 61 and 63 points. Using such a narrow portion of the scale is problematic as minor score variations can result in significant changes in slope. The ability to demonstrate a local slope variation for a narrow band of scores that covers only 5% of the total range of the scale does not mean that the overall relationship is nonlinear.

Senesh and Reinkensmeyer used a moving window of 20 points on the FM-UE to inspect for local variations in proportional recovery slope.^4^ They found that the recovery proportionality varied with baseline FM-UE score, and suggested that the association between impairment and recovery is nonlinear. While this is a stronger case for nonlinearity, local slope variations can arise from random variation and measurement noise. To challenge proportional recovery, the patterns of nonlinearity found in this study should be reproduced in independent FM-UE datasets. Otherwise, if local slope variations are caused by noise rather than true biological variation, fitting a nonlinear function is not advised as it will model nonlinearities that are unlikely to be present in other datasets, resulting in poorer generalisability than linear regression. A recent article by Goldsmith et al. compared linear and nonlinear modelling of bootstrapped empirical recovery data from four published proportional recovery studies.^5-9^ Using cross-validation to apply each model to unseen data, they found that linear modelling performed better than nonlinear modelling, indicating that the relationship between baselines and recovery in the FM-UE is best described by the proportional recovery rule.^5^

A recent study of 1085 patients reported that the recovery proportionality in the FM-LE differed between four groups, based on baseline FM-LE (>17, ≤ 17) and age at stroke onset (≥60, <60).^10^ The groups were obtained via stratified bootstrapping over 1000 iterations, with the baseline FM-LE and age at stroke onset thresholds as partial delimiters. The authors reported that younger age and higher baseline FM-LE were associated with higher recovery proportionalities, citing p<0.001 for the difference in recovery proportion between groups. The article states that a 95% confidence interval was obtained for the mean recovery proportion for each group, however it is not entirely clear which statistical test was used to compare the mean recovery proportions. In our view, the highly statistically significant results for the difference in recovery proportions is not consistent with the data presented in their Figure 4 and Supplemental Table IX, which suggest that the 95% confidence intervals for recovery proportion overlaps considerably between groups.

**Heteroscedasticity of Residuals**

For proportional recovery data, the distribution of residuals often violates the linear regression assumption of homoscedasticity,^3,11^ which can lead to biased standard error estimates.^12^ A recent study found proportional recovery in the FM-LE, but dismissed their findings due to heteroscedasticity.^10^ However, violations of homoscedasticity can be permissible in linear regression models since the slope and intercept remain unbiased, and there exist modified standard error calculations that are robust against heteroscedasticity.^12^ Furthermore, since proportional recovery and baseline-outcome regression have the same residuals, heteroscedasticity will persist even if baseline scores are used to predict outcomes, thus baseline-outcome regression will be just as affected by violations of homoscedasticity as proportional recovery. Alternative types of regression such as generalised least squares or weighted least squares may be helpful when significant heteroscedasticity is detected.

**Hierarchical Clustering for Classifying Fitters and Non-fitters**

Lohse and colleagues raised a concern that hierarchical clustering, commonly used to classify fitters and non-fitters, has a risk of constructing groups that belong to the same underlying distribution and are not meaningfully distinct.^2^ While this is a valid concern, this is a general caveat of hierarchical clustering methods, and is not specific to proportional recovery. As an alternative, neurological biomarkers like motor evoked potential status can be used to classify fitters and non-fitters, who experience meaningfully different upper limb motor recoveries and are functionally distinct.^5,8,9^

**Motor Evoked Potentials for Classifying Fitters and Non-fitters**

Lee et al. argued that motor evoked potential (MEP) status early after stroke is insufficient at classifying proportional recovery non-fitters, citing two studies as evidence that some MEP negative patients can still experience significant upper limb motor recovery.^13,14^ However, while non-fitters do not experience the same proportional recovery as fitters, this does not necessarily mean that non-fitters have zero recovery. Patients who are non-fitters can still experience some recovery in the FM-UE, while also being accurately classified by a MEP negative status. Furthermore, while these studies did observe that MEP negative patients can achieve some degree of motor recovery, these cases only account for about 25% or less of the MEP negative patients. Thus, while there may be some exceptions, a MEP negative status is overall indicative of poor motor recovery capacity after stroke. Overall, MEP status may not be 100% accurate, but is still a good and sufficient predictor of proportional recovery fitters and non-fitters. Non-fitters can still experience a small amount of motor recovery, and the ability for some MEP negative patients to achieve some motor recovery does not preclude the ability for MEP status to be an indicator of proportional recovery fitters and non-fitters.

**Longitudinal Modelling Supports Proportional Recovery**

In van der Vliet et al. FM-UE outcomes after stroke were modelled longitudinally using the following exponential growth function, where *S*_max_ is the maximum score, *S*_baseline_ is the baseline score, *t* is time, *S_t_* is the score at time *t*, and *τ* is a time constant equal to the time required to achieve 63% of the recovery term *β⋅* (*S*_max_ – *S*_baseline_).^15^

$$S_{t}=S_{\mathrm{baseline}}+\beta\cdot\left( S_{\max}-S_{\mathrm{baseline}} \right)\cdot(1-e^{-t/\tau})$$

This approach has been commended for avoiding mathematical coupling,^16^ however the model is fundamentally related to baseline-outcome regression and proportional recovery. For a given value of *t*, the exponential growth function reduces to one of the expressions for baseline-outcome regression (Equation 3, full text). Similarly, *S*_baseline_ can be moved to the left side of the equation, without changing any coefficients, to produce the formula for proportional recovery. Thus, the ability of this longitudinal model to predict outcomes after stroke suggests that proportional recovery is not only a valid model for stroke recovery, but also applicable irrespective of time. Furthermore, the exponential growth function indicates that the rate of recovery after stroke decreases over time, which is entirely consistent with compression towards ceiling and proportional recovery.

**Supplemental References**

1. Hawe RL, Scott SH, Dukelow SP. Taking Proportional Out of Stroke Recovery. *Stroke*. 2019;50(1):204-211. doi:10.1161/STROKEAHA.118.023006

2. Lohse KR, Hawe RL, Dukelow SP, Scott SH. Statistical Considerations for Drawing Conclusions About Recovery. Article. *Neurorehabil Neural Repair*. 2021;35(1):10-22. doi:10.1177/1545968320975437

3. Lee HH, Kim DY, Sohn MK, et al. Revisiting the Proportional Recovery Model in View of the Ceiling Effect of Fugl-Meyer Assessment. Article. *Stroke*. 2021:3167-3175. doi:10.1161/STROKEAHA.120.032409

4. Senesh MR, Reinkensmeyer DJ. Breaking Proportional Recovery After Stroke. Article. *Neurorehabil Neural Repair*. 2019;33(11):888-901. doi:10.1177/1545968319868718

5. Goldsmith J, Kitago T, de la Garza AG, et al. The proportional recovery rule redux Arguments for its biological and predictive relevance. *bioRxiv*. 2021:2021.05.20.445022. doi:10.1101/2021.05.20.445022

6. Zarahn E, Alon L, Ryan SL, et al. Prediction of motor recovery using initial impairment and fMRI 48 h poststroke. *Cereb Cortex*. 2011;21(12):2712-21. doi:10.1093/cercor/bhr047

7. Winters C, van Wegen EE, Daffertshofer A, Kwakkel G. Generalizability of the Proportional Recovery Model for the Upper Extremity After an Ischemic Stroke. *Neurorehabil Neural Repair*. 2015;29(7):614-22. doi:10.1177/1545968314562115

8. Byblow WD, Stinear CM, Barber PA, Petoe MA, Ackerley SJ. Proportional recovery after stroke depends on corticomotor integrity. *Ann Neurol*. 2015;78(6):848-859. doi:10.1002/ana.24472

9. Stinear CM, Byblow WD, Ackerley SJ, Smith MC, Borges VM, Barber PA. Proportional Motor Recovery After Stroke: Implications for Trial Design. *Stroke*. 2017;48(3):795-798. doi:10.1161/strokeaha.116.016020

10. Lee HH, Sohn MK, Kim DY, et al. Understanding of the Lower Extremity Motor Recovery After First-Ever Ischemic Stroke. *Stroke*. 2022;53(10):3164-3172. doi:10.1161/STROKEAHA.121.038196

11. Kundert R, Goldsmith J, Veerbeek JM, Krakauer JW, Luft AR. What the Proportional Recovery Rule Is (and Is Not): Methodological and Statistical Considerations. Article. *Neurorehabil Neural Repair*. 2019;33(11):876-887. doi:10.1177/1545968319872996

12. Schmidt AF, Finan C. Linear regression and the normality assumption. *J Clin Epidemiol*. 2018;98:146-151. doi:10.1016/j.jclinepi.2017.12.006

13. van Kuijk AA, Pasman JW, Hendricks HT, Zwarts MJ, Geurts AC. Predicting hand motor recovery in severe stroke: the role of motor evoked potentials in relation to early clinical assessment. *Neurorehabil Neural Repair*. 2009;23(1):45-51. doi:10.1177/1545968308317578

14. Hendricks HT, Pasman JW, van Limbeek J, Zwarts MJ. Motor evoked potentials in predicting recovery from upper extremity paralysis after acute stroke. *Cerebrovasc Dis*. 2003;16(3):265-71. doi:10.1159/000071126

15. van der Vliet R, Selles RW, Andrinopoulou E-R, et al. Predicting Upper Limb Motor Impairment Recovery after Stroke: A Mixture Model. *Ann Neurol*. 2020;87(3):383-393. doi:10.1002/ana.25679

16. Bowman H, Bonkhoff A, Hope T, Grefkes C, Price C. Inflated Estimates of Proportional Recovery From Stroke: The Dangers of Mathematical Coupling and Compression to Ceiling. *Stroke*. 2021;52(5):1915-1920. doi:10.1161/strokeaha.120.033031
